# Quantification of intracellular nucleotide sugars and formulation of a mathematical model for prediction of their metabolism

**DOI:** 10.1186/1753-6561-5-S8-P10

**Published:** 2011-11-22

**Authors:** Ioscani Jiménez del Val, Judit M Nagy, Cleo Kontoravdi

**Affiliations:** 1 Department of Chemical Engineering, Imperial College London, London, SW7 2AZ, UK; 2 Institute of Biomedical Engineering, Imperial College London, London, SW7 2AZ, UK

## 

The US FDA and the European Medicines Agency have recently proposed the implementation of the Quality by Design (QbD) paradigm to the manufacture of biopharmaceuticals. Its implementation requires the use of all available knowledge of a given product, including the parameters that affect its quality, for the design, optimization and control of the manufacturing process. The goal is to ensure that quality is built into the product at every stage of the manufacturing process. Most licensed monoclonal antibodies (mAbs) are based on the immunoglobulin G isotype and contain a consensus N-linked glycosylation site on the Cγ2 domains of their heavy chains. Studies have found that the oligosaccharides attached to this site dramatically influence the efficacy of mAbs as therapeutics either by reducing their serum half-life or by directly affecting the mechanisms they trigger *in vivo*[1,2], thus defining glycosylation as a critical quality attribute of mAbs under the QbD scope. It has been recently proposed that detailed mathematical models will play a critical role in the design, control and optimization of biopharmaceutical manufacturing processes under the QbD scope [3]. To our knowledge, there are currently no mathematical models that relate mAb glycosylation with cell culture conditions.

Several reports have shown that glycosylation is directly affected by the intracellular availability of nucleotide sugar donors (NSDs) [4] which are the co-substrates for the glycosylation reactions that occur in the Golgi apparatus. During culture, cells synthesize all the relevant NSDs from glucose through the nucleotide sugar metabolic pathway. In an effort to relate process conditions with mAb glycosylation, we have generated a dynamic mathematical model for this metabolic pathway. The NSD pathway described in KEGG [5] was used as the starting point. In the full pathway, four potential carbon sources are converted into the eight main NSDs (UDP-GlcNAc, UDP-Glucose, UDP-Galactose, UDP-GalNAc, UDP-GlcA, GDP-Man, GDP-Fuc and CMP-Neu5Ac) through 31 enzymatic reactions. However, many of the intermediary species are difficult to measure throughout the course of cell culture. For this reason, the kinetic model was reduced based on the methodology described by Nolan and Lee [6] whereby sequential reactions along different branches of the pathway were lumped into single reactions. As an additional simplification, glucose was considered as the only carbon source for the pathway. In order to relate NSD metabolism with macroscopic cell culture variables, a model for cell growth, nutrient depletion, metabolite accumulation and product secretion was formulated based on conventional Monod kinetics. Both models were linked by defining the intracellular glucose accumulation needed for the NSD model as a function of the glucose maintenance energy term (*m_s_*_,_*_glc_*) from the cell culture model; the outlet of NSDs from the cells was associated to the product secretion rate.

**Figure 1:**
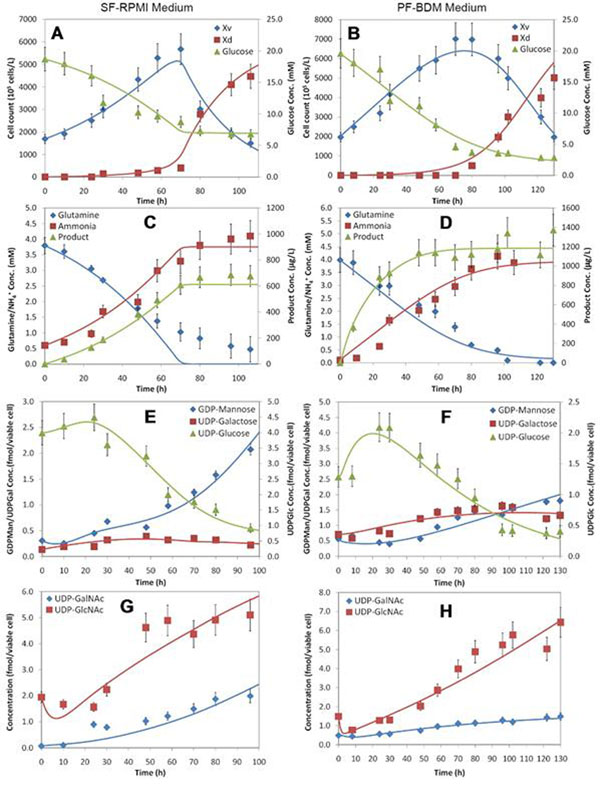
**Model reproduction of the experimental data. A**, **B**, **C** and **D** show reproduction of viable cell counts (Xv), dead cell counts (Xd), glucose, glutamine, ammonia and product concentrations in conventional culture media (SF-RPMI) and optimized chemically defined media (PF-BDM). **E**, **F**, **G** and **H** show reproduction of intracellular NSD concentrations.

In order to estimate the unknown parameters of the combined model, experimental data from Kochanowski and collaborators [7] was used. First, the parameters from the macroscopic model were estimated from the data, including the maintenance energy term for glucose. The results are shown in panels A, B, C and D of Figure 1. Once the cell culture data was reproduced accurately with the estimated parameters, the unknown kinetic parameters from the NSD component of the model were estimated with the intracellular NSD data from Kochanowski et al. [7]. These results are shown in panels E, F, G and H of Figure 1.

Figure 1 shows that, overall, the model reproduces the experimental data accurately. The only exceptions are the UDP-GlcNAc concentration profiles for both culture media and the UDP-GalNAc profiles for the SF-RPMI medium. In the case of UDP-GlcNAc, the model predicts higher accumulation of this NSD towards the end of the data set, whereas the experimental data suggests that the profile flattens out. It is likely that the model overestimates UDP-GlcNAc accumulation because experimental data for CMP-Neu5Ac was unavailable and therefore, this NSD was not considered within the model. From the reduced metabolic network for NSDs, it is known that CMP-Neu5Ac is produced from UDP-GlcNAc. If CMP-Neu5Ac is not considered within the model, it is natural that the model will predict additional accumulation of UDP-GlcNAc. In the case of UDP-GalNAc for the SF-RPMI medium, overaccumulation for this NSD is also predicted by the model. It is possible that this is due to the excess accumulation of UDP-GlcNAc. From the metabolic network, it is known that UDP-GalNAc is directly synthesized from UDP-GlcNAc. If the model predicts higher accumulation of the latter, it will certainly predict higher UDP-GalNAc accumulation as well.

To our knowledge, the model presented in this work is the first to link cell culture variables with intracellular metabolic processes through the glucose maintenance energy term (*m_s_*_,_*_glc_*). Furthermore, it is the first model to relate cell culture variables with intracellular NSD concentrations and the results show that it is capable of reproducing experimental data accurately. However, in order to achieve better reproduction of experimental data and obtain higher confidence in the estimated parameters, additional experimental data is needed. Specifically, the concentration profiles of GDP-Fuc and CMP-Neu5Ac throughout cell culture would lead to improved reproduction of experimental data and predictive capabilities of the model. Furthermore, fed-batch cultures are also necessary to validate the model and its parameters.

Once validated with additional data, our mathematical model for NSD metabolism, can be coupled to a model for Golgi N-linked glycosylation. This combined model would generate a direct link between extracellular glucose concentration, which is a readily measurable process variable, and protein glycosylation. Such a combined model has great potential for the design, control and optimization of manufacturing processes that produce mAbs with *built in* glycosylation-associated quality as proposed under the QbD paradigm.
